# Recent trends in primary-care antidepressant prescribing to children and young people: an e-cohort study

**DOI:** 10.1017/S0033291716002099

**Published:** 2016-09-09

**Authors:** A. John, A. L. Marchant, D. L. Fone, J. I. McGregor, M. S. Dennis, J. O. A. Tan, K. Lloyd

**Affiliations:** 1 Farr Institute of Health Informatics Research, Swansea University Medical School, Singleton Park, Swansea, UK; 2 Division of Population Medicine, School of Medicine, Cardiff University, Cardiff, UK

**Keywords:** Antidepressants, children, depression, prescribing, young people

## Abstract

**Background:**

Concerns relating to increased use of psychotropic medication contrast with those of under-treatment and under-recognition of common mental disorders in children and young people (CYP) across developed countries. Little is known about the indications recorded for antidepressant prescribing in primary care in CYP.

**Method:**

This was an electronic cohort study of routinely collected primary-care data from a population of 1.9 million, Wales, UK. Poisson regression was undertaken to model adjusted counts of recorded depression symptoms, diagnoses and antidepressant prescriptions. Associated indications were explored.

**Results:**

3 58 383 registered patients aged 6–18 years between 1 January 2003 and 31 December 2013 provided a total of 19 20 338 person-years of follow-up. The adjusted incidence of antidepressant prescribing increased significantly [incidence rate ratio (IRR) for 2013 = 1.28], mainly in older adolescents. The majority of new antidepressant prescriptions were for citalopram. Recorded depression diagnoses showed a steady decline (IRR = 0.72) while depression symptoms (IRR = 2.41) increased. Just over half of new antidepressant prescriptions were associated with depression (diagnosis or symptoms). Other antidepressant prescribing, largely unlicensed, was associated with diagnoses such as anxiety and pain.

**Conclusion:**

Antidepressant prescribing is increasing in CYP while recorded depression diagnoses decline. Unlicensed citalopram prescribing occurs outside current guidelines, despite its known toxicity in overdose. Unlicensed antidepressant prescribing is associated with a wide range of diagnoses, and while accepted practice, is often not supported by safety and efficacy studies. New strategies to implement current guidance for the management of depression in CYP are required.

## Introduction

The recognition and management of mental disorders in children and young people (CYP) is increasingly a source of controversy. There are concerns that mental disorders in CYP are not recognized and are undertreated (Department of Health, 2012, 2015) which are in contrast to those in relation to the medicalization of unhappiness and normal human experience with resulting over-diagnosis and over-treatment (Watts, 2012; APA, 2013; Dowrick & Frances, 2013; Gaughwin, 2014). The former is supported by evidence that their conditions are often not appropriately recognized (Fitzpatrick *et al.* 2012) and that when they are recognized there is a serious lack of treatment resources (Royal College of Psychiatrists, 2015). In addition, studies have demonstrated that antidepressants in particular have been underused in CYP who have taken their own lives (Windfuhr *et al.* 2008; Dudley *et al*. 2010). The latter is supported by the already increasing use of psychotropic medication in CYP in the United States (Olfson *et al.* 2002) Europe (Steinhausen & Bisgaard, 2014) and the UK (Middleton *et al.* 2001; Rani *et al.* 2008; Lockhart & Guthrie, 2011; John *et al.* 2015) but may be more of an issue for the United States due to differing diagnostic practices (James *et al.* 2014). Nonetheless mental health and well-being are of increasing public health importance worldwide, with depression estimated to be the leading cause of disease burden by 2030 (WHO, 2004). Studies have shown that depression is common in adolescents with at least one-half of those diagnosed going on to have recurrent depression in adulthood (Kessler *et al.* 2001). The adverse impact of mental health disorders on life outcomes such as educational under-achievement, employment, (Kessler *et al.* 1995) and teenage pregnancy (Kessler *et al.* 1997) plus the serious disruptions to individuals’ lives and those of their families has resulted in calls for increased outreach services and early intervention (WHO, 2004).

The most recent data analysed in the UK, up to 2009, has demonstrated an increase in all new antidepressant prescriptions across all age groups (Middleton *et al.* 2001; Lockhart & Guthrie, 2011; Wijlaars *et al.* 2012). Kendrick *et al.* (2015*a*) examined antidepressant prescribing in those with non-psychotic depression symptoms or diagnoses only across all age groups and found prescribing rates for patients with incident depression fell following the introduction of depression guidelines for adults (NICE, 2004) and the Quality and Outcomes Framework (BMA and NHS Employers, 2006) while rates for recurrent depression increased. A review of the use of selective serotonin reuptake inhibitors (SSRIs) in childhood in 2004 concluded that fluoxetine was the only one with a favourable risk/benefit profile (Whittington *et al.* 2004) and thus it is the only antidepressant licensed for use in CYP (NICE, 2005). Following this review the National Institute for Health and Care Excellence (NICE) published clinical guideline 28 (NICE, 2005) in 2005 (updated March 2015), recommending that the first-line treatment for moderate to severe depression in CYP is psychological therapies. In general, antidepressant medication should be offered only in combination with psychological therapies. If fluoxetine is unsuccessful after an adequate trial at adequate dosages and the depression is sufficiently severe to justify the trial of another antidepressant, NICE recommends citalopram or sertraline as second-line treatments. However, this is required to be logged as unlicensed use. A study using GP practice records up to 2009 demonstrated that citalopram (not fluoxetine) represented the highest annual rate of change in new prescribing for young people in England (Wijlaars *et al.* 2012).

Primary care is often the first point of contact for individuals seeking help for mental health problems. Previous studies utilizing routine data have shown an increase in recording of symptoms alongside a corresponding decrease in the recording of diagnoses for anxiety and depression in adults (Rait *et al.* 2009; Walters *et al.* 2012; Kendrick *et al.* 2015*a*) and CYP (Wijlaars *et al.* 2012; John *et al.* 2015). The decrease in recording of depression diagnoses may be partially attributable to cautious diagnostic behaviour by GPs following the controversy over the safety of antidepressant use in CYP in 2003 [Committee of Safety of Medicines (CSM), 2003] and strategic labelling of depression in order to maintain adherence with Quality Outcomes Framework (QOF) guidelines (Mitchell *et al.* 2011; Wijlaars *et al.* 2012), a pay-for-performance indicator in primary care which includes assessment of depression diagnoses. There is only one previous UK study using 2001 data that attempts to identify the indications associated with the prescription of antidepressants for CYP (Murray *et al.* 2004) using routinely collected primary-care data. This was prior to the 2003 CSM advice, changes in GP recording behaviour and QOF. Given the concerns about over-medicalization of symptoms and that the increase in new prescriptions of antidepressants for CYP is not reflected by a comparative trend in depression diagnosis, a better understanding of the context in which antidepressant use is initiated in this population is warranted. Additionally, an examination of more recent trends in the incidence of the prescribing of antidepressants by age group, particularly fluoxetine and citalopram is required.

## Aims

The aim of this study is to examine recent trends and indications for primary-care prescribing of antidepressants in children and young people (CYP).

## Method

### Design

A retrospective electronic cohort study was conducted utilizing the Secure Anonymized Information Linkage System (SAIL Databank; http://www.saildatabank.com) developed in the Health Information Research Unit (HIRU) in Swansea University Medical School.

### Data source

The SAIL Databank is an expanding data repository (over 2 billion records) of anonymized person-based linkable data to support research. SAIL was established by HIRU at Swansea University in 2004 and forms part of the Health e-Research Collaboration UK (HeRC UK), led by the Medical Research Council (MRC) and based in the Centre for the Improvement of Population Health through e-Records Research (CIPHER). CIPHER is a UK Clinical Research Collaboration (UKCRC) Public Health Research Centre of Excellence set within the Farr Institute at Swansea University Medical School. Policies, structures and controls are in place to protect patient confidentiality. A high-performance computing infrastructure and a reliable matching, anonymization and encryption process in conjunction with the NHS Wales Informatics Service uses a split file approach to import data into SAIL. This ensures anonymization and confidentiality, while maintaining the facility of data linkage at the level of the individual to any of the datasets housed in SAIL, such as general practice records, hospital admissions and demographic information (Ford *et al.* 2009; Lyons *et al.* 2009).

For the purpose of this study data were utilized from several datasets linked at the patient level:

• The Welsh Demographic Service (WDS) is a core dataset available within the SAIL Databank and part of a set of services to manage administrative information (demographic data) for NHS patients in Wales. The WDS was introduced early in 2009 replacing a similar service known as the NHS Wales Administrative Register (NHS AR). The WDS is a register of all individuals who have at some point in time been registered with a GP in Wales or required some form of NHS healthcare provision in Wales.
• The General Practice Database (GPD) contains attendance and clinical information for all general practice interactions including symptoms, investigations, diagnoses and prescribed medication. Currently, regularly updated data are collected from 195 SAIL practices (out of 474 in Wales) covering a total population of over 1.9 million.
• Small-area deprivation scores were taken from the Welsh Index of Multiple Deprivation 2011 (http://gov.wales/statistics-and-research/welsh-index-multiple-deprivation/technical-information/?lang=en). This index is derived from eight separate domains of deprivation including income, employment and education. This dataset assigns a deprivation score to all 1909 Lower Layer Super Output Areas (LSOAs) in Wales, with an average population of 1500 people (range: 998–4402). LSOAs were ranked by deprivation score and divided into quintiles of equal counts.

### Study population and setting

Individuals aged from 6 to 18 years between 1 January 2003 and 31 December 2013 were identified in the WDS. Due to the small number of prescriptions in those aged 0–5 years (<50) this age group was excluded. Data collection began either 6 months from GP registration or at study onset whichever was the later. Data collection ended at the end of registration with a SAIL GP, date of death, 19th birthday or the study end date, whichever was sooner. Individuals supplying a minimum of 6 months of data based on these criteria (and therefore registered with a SAIL GP for a minimum of 1 year) were included in the cohort. Each individual could supply more than one period of data provided the above criteria were met. For each year, data were collected between the start and end dates identified when constructing the original cohort or, between 1 January and 31 December if an individual's period of data collection extended beyond these dates.

### Measures

We queried the primary-care database using db2 structured query language (SQL), implementing Read Codes versions 2 and 3 (5-byte set). Read codes, a hierarchical nomenclature, are used in primary care to record clinical summary information. The Read codes and algorithms being used to identify symptoms or diagnoses of depression have been developed and utilized in previous research and excluded those for psychosis (Supplementary Table S1) (Kessler *et al.* 1997; Whittington *et al.* 2004; Wijlaars *et al.* 2012; John *et al.* 2016). Antidepressant prescriptions were divided into SSRIs, tricyclic antidepressants (TCADs) and other antidepressants (Supplementary Table S2) as listed in the British National Formulary and in use at any time during the study period.

Individuals with an incident or prevalent antidepressant prescription or, incident, prevalent or recurrent diagnosis of depression or symptom of depression were identified in each year of the cohort. Mixed anxiety and depression is reported elsewhere (John *et al.* 2015). An incident episode was defined as no record of the given subtype (antidepressant prescription or depression diagnosis or depression symptom) in the previous 12 months. Participants could have more than one episode recorded across the study period as long a period of at least 12 months existed between entries within that subtype, in keeping with previous studies (Rait *et al.* 2009; Walters *et al.* 2012; Wijlaars *et al.* 2012; John *et al.* 2015). Similarly participants could have more than one subtype recorded within a given year, e.g. a depression diagnosis and antidepressant prescription, again as long a period of at least 12 months existed between entries within that subtype. An annual prevalent episode was defined as an individual with any record of the given subtype (antidepressant prescription or depression diagnosis or depression symptom) in a target year (John *et al.* 2016). An annual recurrent episode was defined as the first record in a given year of a given subtype where a record of that subtype exists previously in that individuals GP record.

We conducted time trend analyses to determine whether significant changes in rates of recorded depression diagnosis or symptom codes occurred following the introduction of NICE guidance in 2004 and 2005 and the introduction of QOF indicators for depression in quarterly blocks, as conducted in previous literature (Kendrick *et al.* 2015*a*). We also conducted a time trend analysis by quarter in the 24 months prior to the United States Food and Drug Administration (August 2011), Safety Communication (United States Food and Drug Administration) followed by a UK Government Drug Safety Update (December 2011), (Medicines and Healthcare products Regulatory Agency, 2011) that considered citalopram was associated with dose-dependent QT interval prolongation of antidepressant subtype to assess any change in prescribing of type of antidepressant.

Routine data does not explicitly link medication prescription with a diagnosis. The records of individuals with a new antidepressant prescription were further analysed to identify the indication for which the medication was prescribed. GP records were reviewed for 1 year before and 6 months after the initial incident prescription date for depression and anxiety diagnoses (including mixed anxiety and depression) and symptoms before searching for other possible indications. After depression and anxiety, the indications searched were: pain, enuresis, attention deficit hyperactivity disorder, conduct disorders, autism, headaches, migraine prophylaxis sleep problems, other codes of interest (including tearfulness and psychosis), irritable bowel syndrome, stress, phobias and obsessive disorders, dissociative disorders and eating disorders (Gardarsdottir *et al.* 2007). If an individual with a new antidepressant prescription had a diagnosis or symptom of depression, anxiety or mixed anxiety depression recorded it was assumed that this was the indication for which the medication was prescribed and they were not examined for any further diagnoses.

Age, gender and deprivation quintile data were collected based upon the onset of data collection for each year. Age was categorized into three groups; 6–10, 11–14 and 15–18 years.

### Statistical analysis

Annual incidence rates were calculated using person-years at risk (PYAR) as the denominator. Annual prevalence rates were also calculated using PYAR as a denominator. This is a more appropriate denominator than the number of registered cases because each individual's duration of follow-up was not fixed (John *et al.* 2016). Poisson regression was undertaken to model the counts of both recorded depression symptoms, diagnoses and antidepressant prescriptions, as a function of year of diagnosis, gender, age group and deprivation. The significance of variables in the Poisson regression modelling was assessed using Wald tests. Robust standard errors for the estimated incidence rate ratios (IRRs) were used to account for clustering within practices. Analysis was conducted using SPSS v. 20 (IBM Corp., USA; syntax available on request).

Quarterly rates were calculated using person-quarters at risk as a denominator, in keeping with previous research of this type (Kendrick *et al.* 2015*b*). An interrupted time-series analysis was undertaken to assess the significance of changes based on quarterly data using segmented regression (Wagner *et al.* 2002). This analysis divides the time series into two periods before and after a given event to assess whether there was a significant step change or change in slope of the line following an event (futher details are available in Kendrick *et al.* 2015*b*). An autoregressive, integrated moving-average time-series model was implemented accounting for autocorrelation.

## Ethical statement

Approval was granted from the HIRU Information Governance Review Panel (IGRP), an independent body consisting of a range of government, regulatory and professional agencies, which oversees study approvals in line with ethical permissions already granted to the analysis of data in the SAIL Databank (Lyons *et al.* 2009; Ford *et al.* 2009). Approval number 0242.

## Results

### Study population

A total of 3 58 383 individuals aged 6–18 years between 1 January 2003 and 31 December 2013 contributed 19 20 338 person-years of data. The mean follow-up time was 5.36 years.

### Trends in the incidence of antidepressant prescribing over time

A total of 10 037 (2.8%) individuals received 10 650 incident antidepressant prescriptions during the study period. Of these 10 650 prescriptions, 9754 [91.6%, 95% confidence interval (CI) 91.1–92.1] represent the first ever record of such a prescription in the GP data. Of those incident prescriptions that had ⩾1 year of GP follow-up data (*n* = 4775), 3298 received ⩾2 prescriptions (69.1%, 95% CI 67.8–70.4) of which 1809 (37.9%, 95% CI 36.5–39.3) received ⩾5 prescriptions in the subsequent year.

**Fig. 1.**
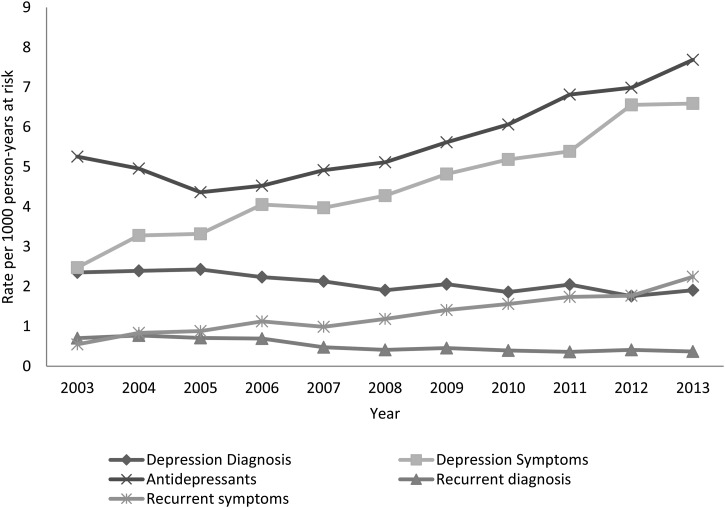
Rate of incident and recurrent depression diagnosis and symptoms and incident antidepressant prescriptions over time.

**Fig. 2.**
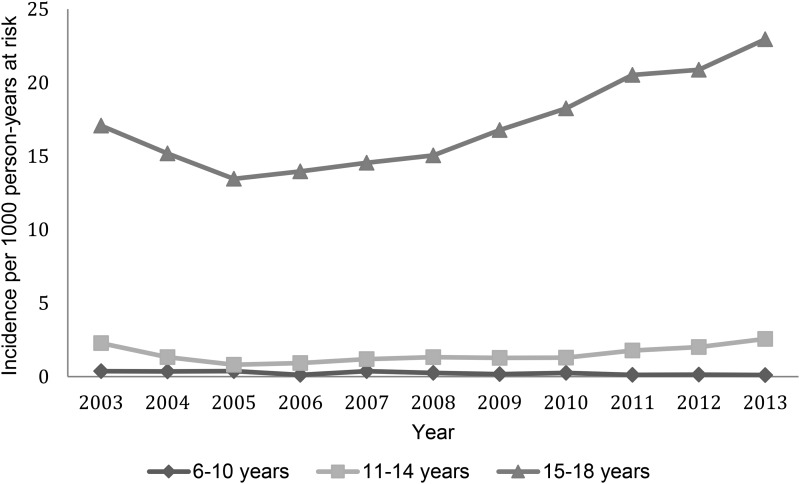
Incidence of antidepressant prescription by age group over time.

The crude incidence of antidepressant prescribing showed an initial decline (Fig. 1) from 5.26 to 4.36 cases/1000 PYAR from 2003 to 2005 (IRR 0.73, 95% CI 0.66–0.80). However, the rate of prescribing began to increase from 2005 onwards to 7.69 cases/1000 PYAR in 2013. Adjusted IRRs for year, gender, age group and deprivation are shown in Table 1. The prescription rate was lower between 2004 and 2008 compared to the baseline year 2003 but increased significantly between 2011 and 2013 (2013: IRR 1.28, 95% CI 1.18–1.40). The prescription rate was higher in females (IRR 2.72, 95% CI 2.60–2.85) and highest in the 15–18 years age group (Fig. 2), with a smaller increase in 11- to 14-year-olds.

**Table 1.** Incidence rate ratios for depression diagnosis, symptoms and antidepressant prescription

	Depression diagnosis		Depression symptoms		Antidepressant prescriptions	
Variable	Events	IRR (95% CI)^a^	Events	IRR (95% CI)^a^	Events	IRR (95% CI)^a^
Gender						
Male	966	Reference	2210	Reference	2976	Reference
Female	3094	3.38 (3.14–3.64)	6267	3.00 (2.86–3.15)	7674	2.72 (2.60–2.85)
Age group, yr						
6–10	28	Reference	191	Reference	178	Reference
11–14	432	18.40 (12.01–28.00)	1636	9.83 (8.30–11.60)	926	5.97 (4.69–7.60)
15–18	3600	170.34 (113.04–256.69)	6650	43.8 (37.34–51.5)	9546	68.3 (54.5–85.5)
Deprivation^b^						
1	1156	Reference	2210	Reference	3260	Reference
2	776	1.17 (1.04–1.32)	1632	1.19 (1.11–1.27)	2177	1.14 (1.06–1.23)
3	726	1.25 (1.12–1.40)	1586	1.27 (1.19–1.36)	2097	1.25 (1.17–1.34)
4	542	1.53 (1.39–1.69)	1200	1.52 (1.42–1.64)	1524	1.50 (1.40–1.62)
5	540	1.99 (1.81–2.19)	1199	1.75 (1.65–1.86)	1579	1.94 (1.81–2.07)
Year						
2003	419	Reference	441	Reference	937	Reference
2004	456	0.91 (0.81–1.02)	625	1.21 (1.10–1.34)	945	0.84 (0.76–0.93)
2005	465	0.92 (0.82–1.03)	636	1.2 (1.06–1.37)	836	0.73 (0.66–0.80)
2006	425	0.83 (0.73–0.94)	772	1.46 (1.34–1.59)	861	0.75 (0.68–0.83)
2007	402	0.79 (0.69–0.90)	750	1.43 (1.27–1.60)	928	0.81 (0.74–0.88)
2008	357	0.70 (0.60–0.82)	801	1.52 (1.40–1.65)	958	0.83 (0.77–0.90)
2009	379	0.75 (0.66–0.86)	887	1.71 (1.54–1.89)	1033	0.91 (0.81–1.02)
2010	332	0.68 (0.56–0.83)	925	1.85 (1.69–2.01)	1082	0.99 (0.91–1.08)
2011	341	0.76 (0.67–0.85)	897	1.94 (1.76–2.13)	1134	1.12 (1.03–1.22)
2012	258	0.65 (0.58–0.74)	962	2.37 (2.16–2.60)	1025	1.15 (1.04–1.28)
2013	226	0.72 (0.62–0.83)	781	2.41 (2.16–2.68)	911	1.28 (1.18–1.40)

IRR, Incidence rate ratio; CI, confidence interval.

a Adjusted for calendar year, gender, age group and deprivation.

b Deprivation: 1 = least deprived; 5 = most deprived.

**Fig. 3.**
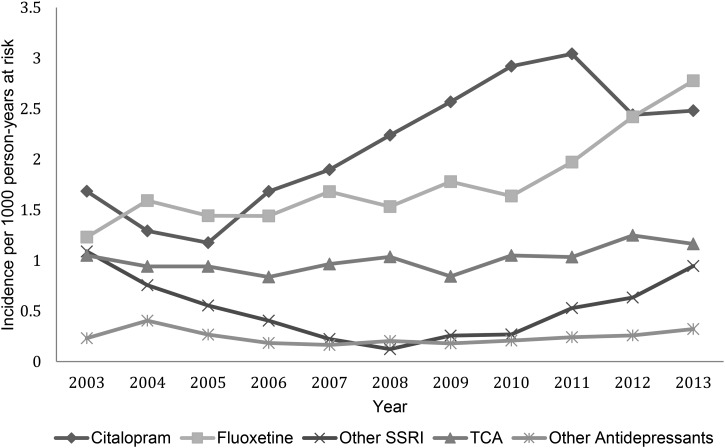
Incidence of antidepressant prescriptions by antidepressant type over time.

SSRIs represented the most frequently prescribed antidepressant across the study period. Citalopram, although unlicensed in this age group, was the most popular SSRI, accounting for around 40% of all new prescriptions, followed by fluoxetine at over one-third (Fig. 3). Of the 4019 citalopram prescriptions issued over the study period to those aged 6–18 years, <1% were given to those aged 6–10 (*n* = 15), 3% were given to those aged 11–14 (*n* = 134) and 96% (*n* = 3870) were given to those aged 15–18. Of the total citalopram prescriptions 29% (*n* = 1162) were given to 18-year-olds. Prescriptions for other SSRIs, TCAs and other antidepressants remained at a low rate.

A non-significant increasing trend in incident antidepressant prescriptions followed the issue of 2004 NICE guidance (Supplementary Fig. S1,  Supplementary Table S3). Similarly a non-significant increasing trend in prescriptions of fluoxetine in 15- to 18-year-olds alongside a decrease in rate of prescribing of citalopram was evident prior to the 2011 safety warnings relating to citalopram. This non-significant decrease in rates of citalopram prescriptions continued following the 2011 warnings (Supplementary Figure S2; Supplementary Table S4).

The annual prevalence of antidepressant prescriptions and depression diagnoses and symptoms are shown in  Supplementary Table S5. For all subtypes (prescriptions, diagnosis and symptoms), the majority of records represent incident as opposed to prevalent cases.

### Time trends in incident and recurrent diagnosis of depression and depressive symptoms

There were 4060 new diagnoses of depression (3860 individuals) and 8477 new cases of depressive symptoms (7931 individuals) during the study period.

New diagnoses of depression (Fig. 1) decreased significantly from 2.35 cases/1000 PYAR in 2003 to 1.91 cases in 2013 (IRR 0.72, 95% CI 0.62–0.83). Recording of depression symptoms more than doubled from 2.48 to 6.59 cases/1000 person-years (IRR 2.41, 95% CI 2.16–2.68) over the study period (Table 1). Recurrent diagnoses of depression (Fig. 1) decreased significantly over time from 0.71 to 0.36 cases/1000 PYAR over the study period (IRR 0.74, 95% CI 0.20–0.74). Recording of recurrent symptoms of depression significantly increased from 0.55 to 2.24 cases/1000 PYAR at over the study period (IRR 3.60, 95% CI 2.81–4.61) (Supplementary Table S6).

An increasing trend in depression symptoms alongside a decrease in diagnoses is evident (Supplementary Fig. S3) in quarterly time points over the 24 months either side of the issue of 2006 QOF performance indicators for depression. However, this was not statistically significant (Supplementary Table S7). Similarly a non-significant increase in the rate of incident and recurrent depression diagnoses and symptoms can be seen following the 2008 recession in 15- to 18-year-olds (Supplementary Fig. S4,  Supplementary Table S8).

### Age, gender and deprivation

A record of a depression diagnosis, depression symptom(s) and new antidepressant prescriptions were approximately three times more likely in females (diagnosis: IRR 3.38, 95% CI 3.14–3.64; symptoms: IRR 3.00, 95% CI 2.86–3.15; antidepressants: IRR 2.72, 95% CI 2.60–2.85) compared to males (Table 1). This gender difference became more apparent with increasing age. In those aged 6–10 years the female: male ratio for depression diagnosis, symptoms and antidepressant prescriptions were 0.56:1, 1.01:1 and 0.63:1, compared to the female:male ratios of 3.32:1, 2.95:1 and 2.76:1, respectively, in those aged 15–18 years. A record of a depression diagnosis, depression symptom(s) and new antidepressant prescriptions were approximately twice as common in the most deprived compared to the least deprived areas (diagnosis: IRR 1.99, 95% CI 1.81–2.19; symptoms: IRR 1.75, 95% CI 1.65–1.86; antidepressants: IRR 1.94, 95% CI 1.81–2.07) (Table 1).

In keeping with recording of incident depression, recording of both recurrent depression diagnoses and symptoms were more than four times as high in females than males (diagnosis: IRR 4.45, 95% CI 3.85–5.14; symptoms: IRR 4.16, 95% CI 3.76–4.60). Recording of both recurrent depression diagnoses was also significantly higher in the most deprived compared with the least deprived areas (diagnosis: IRR 1.74, 95% CI 1.47–2.05; symptoms: IRR 2.02, 95% CI 1.78–2.31) (Supplementary Table S6).

### Indications for antidepressant prescriptions

**Fig. 4.**
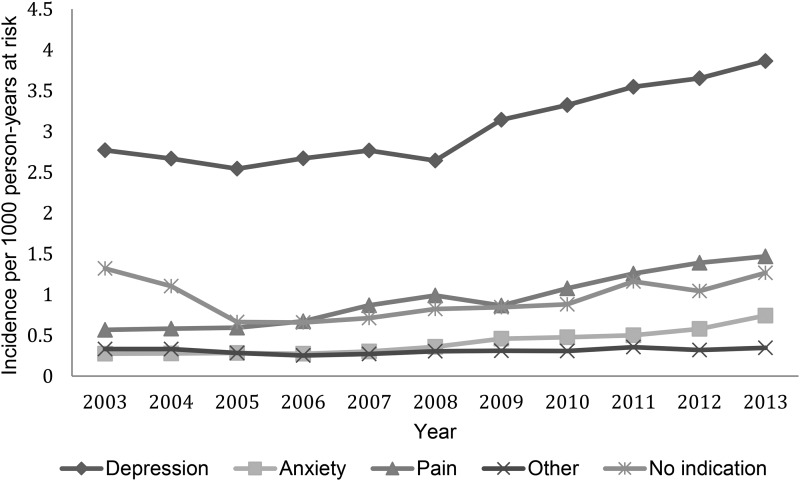
New antidepressant prescriptions by indication over time.

The incidence rates of antidepressant prescriptions by indication are shown in Fig. 4. The biggest single indication for new antidepressant prescriptions was depression (diagnoses and symptoms), which was associated with over one-half of new prescriptions. New prescriptions associated with depression increased from 2.77 cases/1000 person-years in 2003 to 3.86/1000 person-years in 2013. New prescriptions associated with anxiety were less frequent but increased from 0.28 in 2003 to 0.74 cases/1000 person-years in 2013. The incidence of antidepressant prescriptions associated with physical pain also increased over the study period from 0.57/1000 person-years in 2003 to 1.47/1000 person-years in 2013. No other single indication reached sufficient numbers to be shown separately, for example obsessive compulsive disorder was recorded in total <20 times/year over the study period. As a group, prescriptions associated with other indications changed little from 0.33 to 0.35 cases/1000 person-years. Overall, the indication for which an antidepressant was prescribed could not be found for 1793 (16.8%, 95% CI 16.1–17.5) of new prescriptions. New prescriptions for which no indication could be identified showed an initial decline from 1.32 to 0.66 cases/1000 person-years from 2003 to 2006. However, the incidence began to rise again from 2007 onwards reaching 1.27 cases/1000 person-years in 2013. This pattern was reflective of the overall trend for SSRIs over the study period.

## Discussion

### Main findings

The incidence of antidepressant prescriptions for CYP initially decreased between 2003 and 2005 then steadily increased over the study period to the highest incidence in 2013. This lends credence to suggestions that concerns in 2003 (Committee of Safety of Medicines, 2003) over the safety of antidepressant use in CYP waned (Wijlaars *et al.* 2012). Since 2003, the increase in recording of depressive symptoms in CYP has continued throughout the study period although the recording of a diagnosis of depression has shown a small but steady decline. Unlicensed antidepressant prescribing was associated with a range of diagnoses as well as for depression, such as anxiety and pain. Potential indications for antidepressant therapy were found for approximately 83% of new prescriptions.

The increase in prescribing of antidepressants over time is mainly seen in those aged 15–18 years rather than younger age groups. No increase in antidepressant prescribing was found in 6- to 10-year-olds. Citalopram is the most frequently prescribed antidepressant across the study period despite fluoxetine being recommended as first-line treatment and the only antidepressant licensed for use in this age group since it is the only SSRI with a favourable risk/benefit profile (Whittington *et al.* 2004; NICE, 2005, updated March 2015). However, fluoxetine does appear to have increased in popularity from 2009 onwards, with its incidence at a similar level to citalopram by 2013. This is still not consistent with current NICE guidance and advice in the BNF for children. The decline in citalopram prescribing seen from 2011 may have followed a United States Food and Drug Administration (August 2011), Safety Communication (United States Food and Drug Administration) followed by a UK Government Drug Safety Update (December 2011), (Medicines and Healthcare products Regulatory Agency, 2011) that considered citalopram was associated with dose dependent QT interval prolongation. Although we found a downward trend we did not find significant evidence for this. Where data were available, it appears that around 69% of new prescriptions of antidepressants are associated with at least one other prescription being issued in the subsequent year.

### Strengths and limitations

The main strength of this study is the large population sample size of all children and young people in Wales and 11 years of follow-up. There is no reason to believe the results would differ for the entire population of UK children.

The results of the current study reflect trends in the recording of presentations to primary care, and the recognition and treatment by GPs. All prescriptions issued in primary care are recorded within the GPD, but we have no available method of ascertaining from the SAIL Databank if they were dispensed or taken. The incidence rates for diagnoses and symptoms presented are likely to be an underestimate of the true incidence in the community as a whole since those who do not present to their GP, or those with whom symptoms are discussed but not recorded, will be missed by the use of routinely collected data. This is a common feature of all database studies, (Rait *et al.* 2009; Wijlaars *et al.* 2012; Walters *et al.* 2012).

Although we explored a more extensive list of indications for which an antidepressant may be prescribed than previously reported, (Murray *et al.* 2004) the indication for which a medication is prescribed is not explicitly recorded in routinely collected primary-care data. Thus the indications can only be inferred and not conclusively determined. Analyses reported relating to indication are, in essence, a measure of coding behaviour rather than true indications for prescribing. Only those patients with relevant Read codes in their patient record were detected. The method used will give an indication of trends over time but may have resulted in an inflated number of individuals for whom no indication could be found, particularly where a historical record was only made once or where an individual was not registered with a SAIL practice at the time when a diagnosis was recorded. This is a common limitation of all studies of this type (Murray *et al.* 2004). It is possible that GPs consider that the prescribing of an antidepressant implies the diagnosis.

The results of this analysis should not be used to make comparisons of rates of prescribing found across different study populations such as those in the THIN database (Whittington *et al.* 2004). This is because these studies provide a crude non-standardized estimate of the incidence of antidepressant prescribing without taking into account any differences between study populations based on age, gender and deprivation that are known to be associated with levels of childhood depression. Our findings may be influenced by a period effect as the CYP investigated age through the study period. The large proportion of individuals compared to events (e.g. 4060 new depression diagnoses compared to 3860 individuals) and prescriptions (e.g. 10 037 individuals received 9754 incident antidepressant prescriptions) suggests that largely different cohorts of children were assessed across the study period.

It would have been preferable to replicate previous work and examine rates of treated incident, first ever and recurrent depression in relation to issuing of NICE guidance for depression in 2004 and 2005 as done by Kendrick *et al.* (2015*b*) in adults. However, the number of cases of CYP are lower. This smaller sample size combined with the impact of only examining those with adequate follow-up data to determine treatment meant that there were not sufficient numbers to draw meaningful conclusions. Incident antidepressant prescriptions were examined as an alternative.

A further limitation is the lack of information regarding whether and what interventions have been received at secondary and tertiary mental healthcare levels for children and adolescents with depression, i.e. where fluoxetine or citalopram has been initiated at a secondary care level or access to psychological therapies.

The Read codes and algorithms employed in this study have been previously examined against adult survey data (John *et al.* 2016) giving a distinct advantage over other studies of this type where an examination of real-world recording behaviour has not been possible, (Kessler *et al.* 1997; Whittington *et al.* 2004; Wijlaars *et al.* 2012). Further work is required to extend this linkage of routinely collected data to survey data collected on CYP.

### Comparison with previous literature

This study adds to a growing literature demonstrating an increase in psychotropic prescribing for CYP across western cultures (Olfson *et al.* 2002; Dowrick & Frances, 2013; Steinhausen & Bisgaard, 2014). We found the percentage of the CYP population receiving new prescriptions for antidepressants was in keeping with the THIN study from 2009 (Wijlaars *et al.* 2012). The results of this study support and extend previous research demonstrating an increase in recording of depressive symptoms and decrease in depression diagnoses over time (Whittington *et al.* 2004; NICE, 2005; Rait *et al.* 2009; Walters *et al.* 2012; Wijlaars *et al.* 2012). In keeping with previous research (Kendrick *et al.* 2015*a*), no significant trend over time was found in recording of depression diagnosis or symptoms when analysing quarterly time points in the 24 months either side of the 2006 QOF performance indicators for depression or in relation to the 2008 recession in 15- to 18-year-olds. Similarly the non-significant increasing trend in incident antidepressant prescriptions seen following the issue of 2004 and 2005 NICE guidance is in keeping with previous research (Kendrick *et al.* 2015*b*) where no significant change was found in both recurrent and overall episodes of depression treated with antidepressants related to NICE guidance. The positive results reported in this study refer to the number of incident episodes of depression treated following the issue of the guidance.

A previous study from 2001, prior to CSM advice, found indications for around 70% of antidepressant prescriptions, (Murray *et al.* 2004) and did not limit its analysis to new prescriptions. Pain, which accounted for 16% of new prescriptions in the current study, was not examined. The comprehensive list of indications employed in the current study has identified potential indications for 83% of new prescriptions. Many of these indications are unlicensed usage such as for pain. In keeping with previous research, (Murray *et al.* 2004) depression is the most commonly associated indication.

### Implications

It may be useful to issue further guidance and support to GPs in relation to indications for effective antidepressant prescribing and to promote the recording of a diagnosis when issuing a prescription. Tricyclic antidepressants rather than SSRIs are the mainstay of neuropathic pharmacological pain treatment and are considered to be first-line drugs in adult practice (Gregoire & Finley, 2013). They are also used off-license in paediatrics, but without much published evidence. A Cochrane review (Kaminski *et al*. 2011) failed to support their effectiveness in treating CYP's chronic pain. There are currently no published guidelines for neuropathic pain in children (Gregoire & Finley, 2013). Many GPs feel poorly equipped to manage young people's emotional distress and feel there is a lack of clarity (Roberts *et al.* 2013). There are no specific NICE guidelines available for the management of anxiety in CYP. There are NICE guidelines for anxiety (which relate principally to adults; NICE, 2011), depression for CYP (which includes mixed anxiety and depression; NICE, 2005, updated 2015) and for the assessment and treatment of social anxiety disorder (NICE, 2013). These all highlight some considerations for the management of anxiety in CYP. Psychological therapies should be the first line of treatment. Pharmacotherapy should not be routinely offered to treat social anxiety disorder in young people.

Additionally, strategies to improve the implementation of current guidance on the prescribing of antidepressants for depression in CYP should be adopted. This could include adoption of programmes such as Therapeutic identification of depression in young people (TIDY) which trains primary-care staff to differentiate between emotional turmoil and depression and to implement appropriate management strategies (Kramer *et al.* 2012). A current focus should be the use of citalopram rather than fluoxetine as a first line treatment. The updated NICE guidance of 2015 recommends that citalopram should only be prescribed if fluoxetine is unsuccessful or is not tolerated because of side-effects and then only once advice is sought from a senior child and adolescent psychiatrist. These preferences in initiating antidepressant treatment also warrant further research. It may be that GPs find that citalopram is better tolerated than fluoxetine, or results may simply reflect trends seen in adult populations (Kessler *et al.* 2001) despite citalopram's higher case-fatality in overdose than other SSRIs (Hawton *et al.* 2010). It is also possible that the increase in citalopram prescriptions is related to diagnoses other than depression, such as pain, prescribing for which appears to have increased during the study period. The absence of data regarding any interventions that may have been received at secondary and tertiary mental healthcare levels make it difficult to draw conclusions on whether this trend is a reflection of GP preference or of prescribing patterns initiated in secondary or tertiary care. However, the rate of rejection of referrals from primary-care to specialist mental health services (Hinrichs *et al.* 2012) make it likely that treatment for at least a proportion of individuals is exclusive to primary care. Further research is required to fully understand the increasing trend in prescribing without an associated indication. It may be that indications go beyond those studied here or that GPs are increasingly choosing not to record depression in this population.

Over one-half of new antidepressant prescribing was associated with a symptom or diagnosis of depression. New prescriptions associated with depression (diagnosis and symptoms) increased over the study period. This may reflect increased access to treatment and a positive shift towards helping individuals with mental disorder at a younger age or an increased tendency to prescribe medication, particularly where psychological or other treatment options are limited or not available in primary care. This may be a particular problem in more deprived areas where the incidence is nearly double that in more affluent areas. The increasing levels of those receiving antidepressant treatment at 15–18 years of age also highlights the importance of strengthening primary-care mental health care services and supporting the transition to adult mental health services, when required, given the persistence of adolescent mental health problems into adulthood (Rait *et al.* 2009).

A recent mixed methods study has found that general practice referrals to Child and Adolescent Mental Health Services (CAMHS) were three times more likely to be rejected than referrals from all other sources combined (Hinrichs *et al.* 2012). As a result, primary care may remain the most common source of care for CYP with mental health problems, independent of whether a GP feels an individual requires more specialist support. Concerns remain over the safety of many psychotropic medications for CYP, particularly with regards to antidepressants where there has been controversy regarding increased risk of suicidal ideation and attempt following initiation of antidepressant treatment (Committee of Safety of Medicines, 2003; NICE, 2005). The uncertainty over whether those treated but not diagnosed are followed-up in line with QOF guidance (Rait *et al.* 2009) makes the decrease in recording of diagnoses alongside an increase in new prescriptions a cause for concern in this age group.

Overall, the results suggest that GPs are increasingly using non-specific symptom terms for recording depressive mental disorders for CYP. This decrease in recording of diagnoses may be partially attributable to increasingly cautious diagnostic behaviour by GPs. However this behaviour may also be in part due to the revised QOF guidance, (Walters *et al.* 2012). Our results support the reports from GPs on the use of alternative terms and strategic labelling of depression to maintain adherence with guidelines (Rait *et al.* 2009).

Awareness that GPs are increasingly using non-specific symptoms codes rather than formal diagnoses for both adults, (Kessler *et al.* 1997; Wijlaars *et al.* 2012) and CYP, (Whittington *et al.* 2004) across both England and Wales is important for future research based on routinely collected data. New research should examine the impact of a formal diagnosis on patient care e.g. the impact of treatment with or without a diagnosis on the frequency of follow-up or long-term physical and mental health. This is particularly relevant to CYP where care received may have an enduring impact into adulthood (Watts, 2012; Dowrick & Frances, 2013; Gaughwin, 2014).

## Conclusion

This study contributes to a growing debate over increasing rates of psychotropic medication prescriptions in CYP. Antidepressant prescribing, associated with a broad range of indications, is increasing in CYP while the recorded diagnosis of depression shows a steady decline. Citalopram continues to be prescribed as an initial medication outside current guidelines. Unlicensed antidepressant prescribing is associated with a range of diagnoses other than depression, such as anxiety and pain and, whilst accepted practice included in prescribing guidance and advice, it is not supported by safety and efficacy studies, and could be seen as contributing to the over medicalization of CYP. It may be useful to issue guidance and support to GPs in relation to indications for effective antidepressant prescribing and to promote the recording of a diagnosis when issuing a prescription. New strategies to implement current guidance for the management of depression in this population are required.

## Supplementary material

For supplementary material accompanying this paper visit http://dx.doi.org/10.1017/S0033291716002099. click here to view supplementary material
