# Supplementary material for: Recent trends in primary-care antidepressant prescribing to children and young people: an e-cohort study
Source: Psychol Med. 2016 Sep 9;46(16):3315–27. doi: 10.1017/S0033291716002099 (PMC5122314; doi:10.1017/S0033291716002099)
Supplement: Supplementary file 1 [file S0033291716002099sup.zip › S0033291716002099sup001/S0033291716002099sup001.docx]

Supplementary Figure S1. Incident antidepressant prescriptions in relation to 2004 NICE guidance

Guidance for children

Guidance for adults
