# Supplementary material for: Recent trends in primary-care antidepressant prescribing to children and young people: an e-cohort study
Source: Psychol Med. 2016 Sep 9;46(16):3315–27. doi: 10.1017/S0033291716002099 (PMC5122314; doi:10.1017/S0033291716002099)
Supplement: Supplementary file 1 [file S0033291716002099sup.zip › S0033291716002099sup001/S0033291716002099sup005.docx]

Supplementary Table S1. Read codes used to determine GP record of symptoms and diagnosis of depression

| **Category** | **Read Code** | **Text Description** |
| --- | --- | --- |
| Depression Diagnosis | Eu32. | [X]Depressive episode |
|  | Eu320 | [X]Mild depressive episode |
|  | Eu321 | [X]Moderate depressive episode |
|  | Eu322 | [X]Severe depressive episode without psychotic symptoms |
|  | Eu324 | [X]Mild depression |
|  | Eu32y | [X]Other depressive episodes |
|  | Eu32z | [X]Depressive episode, unspecified |
|  | Eu33. | [X]Recurrent depressive disorder |
|  | Eu330 | [X]Recurrent depressive disorder, current episode mild |
|  | Eu331 | [X]Recurrent depressive disorder, current episode moderate |
|  | Eu332 | [X]Recurrent depressive disorder, current episode severe without psychotic symptoms |
|  | Eu334 | [X]Recurrent depressive disorder, currently in remission |
|  | Eu33y | [X]Other recurrent depressive disorders |
|  | Eu33z | [X]Recurrent depressive disorder, unspecified |
|  | Eu341 | [X]Dysthymia |
|  | E118. | Seasonal affective disorder |
|  | E135. | Agitated depression |
|  | E2B.. | Depressive disorder NEC |
|  | E2B1. | Chronic depression |
|  | E291. | Prolonged depressive reaction |
|  | E204. | Neurotic depression reactive type |
|  | E2B0. | Postviral depression |
|  | E112. | Single major depressive episode |
|  | E1120 | Single major depressive episode, unspecified |
|  | E1121 | Single major depressive episode, mild |
|  | E1122 | Single major depressive episode, moderate |
|  | E1123 | Single major depressive episode, severe, without psychosis |
|  | E1125 | Single major depressive episode, partial or unspecied remission |
|  | E1126 | Single major depressive episode, in full remission |
|  | E112z | Single major depressive episode NOS |
|  | E113. | Recurrent major depressive episode |
|  | E1130 | Recurrent major depressive episodes, unspecified |
|  | E1131 | Recurrent major depressive episodes, mild |
|  | E1132 | Recurrent major depressive episodes, moderate |
|  | E1133 | Recurrent major depressive episodes, severe, no psychosis |
|  | E1135 | Recurrent major depressive episodes, partial/unspecified remission |
|  | E1136 | Recurrent major depressive episodes, in full remission |
|  | E1137 | Recurrent depression |
|  | E113z | Recurrent major depressive episode NOS |
| Depression symptoms | 1B17. | Depressed |
|  | 1B1U. | Symptoms of depression |
|  | 1BQ.. | Loss of capacity for enjoyment |
|  | 1BT.. | Depressed mood |
|  | 1BU.. | Loss of hope for the future |
|  | 2257 | O/E – depressed |
