# Supplementary material for: Recent trends in primary-care antidepressant prescribing to children and young people: an e-cohort study
Source: Psychol Med. 2016 Sep 9;46(16):3315–27. doi: 10.1017/S0033291716002099 (PMC5122314; doi:10.1017/S0033291716002099)
Supplement: Supplementary file 1 [file S0033291716002099sup.zip › S0033291716002099sup001/S0033291716002099sup006.docx]

Supplementary Table S2. Read codes used to determine antidepressant prescription

| **Sub-type** | **Read Code** | **Text Description** |
| --- | --- | --- |
| SSRIs | da3.. | FLUVOXAMINE |
|  | da4.. | FLUOXETINE |
|  | da5.. | SERTRALINE |
|  | da6.. | PAROXETINE |
|  | da9.. | CITALOPRAM |
|  | daC.. | ESCITALOPRAM |
| Tricyclics | d71.. | AMITRIPTYLINE HYDROCHLORIDE [ANTIDEPRESSANT] |
|  | d72.. | *BUTRIPTYLINE |
|  | d73.. | CLOMIPRAMINE HYDROCHLORIDE |
|  | d74.. | DESIPRAMINE HYDROCHLORIDE |
|  | d75.. | DOSULEPIN HYDROCHLORIDE |
|  | d76.. | DOXEPIN |
|  | d77.. | IMIPRAMINE HYDROCHLORIDE [ANTIDEPRESSANT] |
|  | d78.. | IPRINDOLE |
|  | d79.. | LOFEPRAMINE |
|  | d7a.. | MAPROTILINE HYDROCHLORIDE |
|  | d7b.. | MIANSERIN HYDROCHLORIDE |
|  | d7c.. | NORTRIPTYLINE |
|  | d7d.. | PROTRIPTYLINE HYDROCHLORIDE |
|  | d7e.. | TRAZODONE HYDROCHLORIDE |
|  | d7f.. | TRIMIPRAMINE |
|  | d7g.. | VILOXAZINE HYDROCHLORIDE |
|  | d7h.. | AMOXAPINE |
|  | d91.. | COMPOUND ANTIDEPRESSANTS A-Z |
|  | d911. | *LIMBITROL 5 capsules ---Discontinued |
|  | d912. | *LIMBITROL 10 capsules ---Discontinued |
|  | d913. | *MOTIPRESS tablets x28CP ---Discontinued |
|  | d914. | *MOTIVAL tablets ---Discontinued |
|  | d916. | TRIPTAFEN tablets -- only one not discontinued? |
|  | d917. | *TRIPTAFEN-M tablets ---Discontinued |
| Other Antidepressants | d8... | MONOAMINE-OXIDASE |
|  | d81.. | PHENELZINE |
|  | d82.. | *IPRONIAZID |
|  | d83.. | ISOCARBOXAZID |
|  | d84.. | TRANYLCYPROMINE |
|  | d85.. | MOCLOBEMIDE |
|  | da1.. | FLUPENTIXOL |
|  | da2.. | TRYPTOPHAN |
|  | da7.. | VENLAFAXINE |
|  | daA.. | REBOXETINE |
|  | daB.. | MIRTAZAPINE |
|  | daD.. | AGOMELATINE |
|  | gde.. | DULOXETINE |
