# Supplementary material for: Recent trends in primary-care antidepressant prescribing to children and young people: an e-cohort study
Source: Psychol Med. 2016 Sep 9;46(16):3315–27. doi: 10.1017/S0033291716002099 (PMC5122314; doi:10.1017/S0033291716002099)
Supplement: Supplementary file 1 [file S0033291716002099sup.zip › S0033291716002099sup001/S0033291716002099sup007.docx]

Supplementary Table S3. Time trend analysis of incident prescriptions of antidepressants in relation to 2004 NICE guidance

| Incidence rate of antidepressant prescription | Estimate of Effects (95% CI) | p- value |
| --- | --- | --- |
| Secular trend | -0.01(-0.04-0.03) | 0.71 |
| Change in level in Q2 2005 | -0.11(-0.38-0.16) | 0.38 |
| Change in level in Q3 2005 | -0.11(-0.38-0.17) | 0.43 |
| Change in level in Q4 2005 | -0.10(-0.40-0.20) | 0.48 |
| Change in level in Q1 2006 | -0.10(-0.43-0.24) | 0.55 |
| Change in level in Q3 2006 | -0.09(-0.50-0.33) | 0.66 |
| Change in level in Q1 2007 | -0.08(-0.59-0.44) | 0.75 |

Supplementary Table S4. Time trend analysis of the rate of prescriptions of fluoxetine and citalopram in relation to 2011 UK safety warnings

| Incidence rate of prescriptions | Estimate of Effects (95% CI) | p- value |
| --- | --- | --- |
| **Fluoxetine** |  |  |
| Secular trend | 0.01(0.00-0.03) | 0.04 |
| Change in level in Q1 2012 | 0.05(-0.06-0.15) | 0.33 |
| Change in level in Q2 2012 | 0.06(-0.05-0.16) | 0.28 |
| Change in level in Q3 2012 | 0.06(-0.05-0.17) | 0.25 |
| Change in level in Q4 2012 | 0.07(-0.05-0.19) | 0.23 |
| Change in level in Q2 2013 | 0.08(-0.06-0.22) | 0.24 |
| Change in level in Q4 2013 | 0.10(-0.07-0.27) | 0.26 |
| **Citalopram** |  |  |
| Secular trend | 0.00(-0.02-0.02) | 0.77 |
| Change in level in Q1 2012 | -0.12(-0.27-0.03) | 0.12 |
| Change in level in Q2 2012 | -0.13(-0.28-0.03) | 0.09 |
| Change in level in Q3 2012 | -0.14(-0.30-0.02) | 0.09 |
| Change in level in Q4 2012 | -0.15(-0.33-0.03) | 0.09 |
| Change in level in Q4 2013 | -0.19(-0.45-0.07) | 0.13 |
| Change in level in Q2 2013 | -0.17(-0.39-0.04) | 0.11 |
|  |  |  |

Supplementary Table S5. Annual prevalence^a^ rate per 1000 person years at risk of depression diagnosis, symptoms and antidepressant prescriptions over the study period

| Year | Antidepressant | | Depression Diagnosis | | Depression Symptoms | |
| --- | --- | --- | --- | --- | --- | --- |
|  | Events | Prevalence (95%CI) | Events | Prevalence (95%CI) | Events | Prevalence (95%CI) |
| 2003 | 1287 | 7.22 (6.84-7.63) | 453 | 2.54 (2.31-2.79) | 460 | 2.58 (2.35-2.83) |
| 2004 | 1418 | 7.44 (7.06-7.84) | 494 | 2.59 (2.37-2.83) | 669 | 3.51 (3.25-3.79) |
| 2005 | 1233 | 6.44 (6.08-6.81) | 505 | 2.64 (2.41-2.88) | 677 | 3.53 (3.27-3.81) |
| 2006 | 1242 | 6.53 (6.17-6.9) | 455 | 2.39 (2.18-2.62) | 826 | 4.34 (4.05-4.65) |
| 2007 | 1278 | 6.77 (6.41-7.15) | 419 | 2.22 (2.01-2.44) | 785 | 4.16 (3.87-4.46) |
| 2008 | 1334 | 7.12 (6.75-7.52) | 379 | 2.02 (1.83-2.24) | 841 | 4.49 (4.19-4.81) |
| 2009 | 1459 | 7.93 (7.53-8.35) | 394 | 2.14 (1.94-2.36) | 945 | 5.14 (4.82-5.48) |
| 2010 | 1591 | 8.92 (8.49-9.37) | 346 | 1.94 (1.74-2.16) | 993 | 5.57 (5.23-5.92) |
| 2011 | 1626 | 9.77 (9.3-10.26) | 355 | 2.13 (1.92-2.37) | 962 | 5.78 (5.42-6.16) |
| 2012 | 1580 | 10.77 (10.24-11.31) | 272 | 1.85 (1.64-2.09) | 1019 | 6.94 (6.52-7.38) |
| 2013 | 1419 | 11.97 (11.36-12.61) | 236 | 1.99 (1.75-2.26) | 850 | 7.17 (6.7-7.67) |
| 1. a. Prevalence calculated as number of individuals with any record of a given subtype recorded in the target year as rate per 1000 person years at risk | | | | | | |

Supplementary Table S6. Incidence rate ratios (IRR) for recurrent depression diagnosis and symptoms

| **Variable** | | **Depression Diagnosis** | | **Depression Symptoms** | |
| --- | --- | --- | --- | --- | --- |
|  |  | **Events** | **IRR(95% CI)^a^** | **Events** | **IRR(95% CI)^a^** |
| Gender | **Male** | 196 | 1.00(1.00-1.00) | 486 | 1.00(1.00-1.00) |
|  | **Female** | 830 | 4.45(3.85-5.14) | 1914 | 4.16(3.76-4.6) |
| Age Group | **6-10** | <5 | 1.00(1.00-1.00) | 10 | 1.00(1.00-1.00) |
|  | **11-14** | 64 | 24.43(7.68-77.73) | 259 | 29.82(14.98-59.36) |
|  | **15-19** | 959 | 411.1(133.8-1263.08) | 2131 | 267.61(135.94-526.79) |
| Deprivation^c^ | **1** | 294 | 1(1.00-1.00) | 698 | 1(1.00-1.00) |
|  | **2** | 207 | 1.25(1.04-1.49) | 526 | 1.25(1.07-1.47) |
|  | **3** | 196 | 1.15(0.94-1.41) | 505 | 1.48(1.29-1.70) |
|  | **4** | 167 | 1.40(1.17-1.67) | 342 | 1.79(1.57-2.05) |
|  | **5** | 159 | 1.74(1.47-2.05) | 324 | 2.02(1.78-2.31) |
| Year | **2003** | 126 | 1(1.00-1.00) | 98 | 1(1.00-1.00) |
|  | **2004** | 147 | 0.97(0.78-1.20) | 159 | 1.35(1.05-1.73) |
|  | **2005** | 136 | 0.88(0.70-1.11) | 169 | 1.41(1.11-1.78) |
|  | **2006** | 131 | 0.84(0.67-1.05) | 214 | 1.78(1.40-2.26) |
|  | **2007** | 90 | 0.58(0.46-0.74) | 187 | 1.56(1.22-2.01) |
|  | **2008** | 77 | 0.50(0.39-0.64) | 222 | 1.84(1.42-2.39) |
|  | **2009** | 84 | 0.55(0.42-0.71) | 259 | 2.19(1.71-2.79) |
|  | **2010** | 71 | 0.48(0.37-0.62) | 279 – 278 | 2.43(1.93-3.05) |
|  | **2011** | 60 | 0.43(0.33-0.57) | 289 - 288 | 2.72(2.15-3.44) |
|  | **2012** | 60 | 0.50(0.39-0.64) | 260 | 2.81(2.25-3.51) |
|  | **2013** | 44 | 0.46(0.28-0.74) | 266 | 3.6(2.81-4.61) |
| a. Adjusted for calendar year, gender, age and deprivation  b. P based on Wald test  c. Deprivation: 1 = least deprived; 5 = most deprived | | | | | |

Supplementary Table S7. Time trend analysis of recording of incident depression diagnosis and symptoms in relation to the 2006 QOF indicators

| Recording of incident depression | Estimate of Effects (95% CI) | p- value |
| --- | --- | --- |
| **Depression diagnosis** |  |  |
| Secular trend | 0.01(-0.01-0.02) | 0.32 |
| Change in level in Q3 2006 | -0.12(-1.25-1.01) | 0.43 |
| Change in level in Q4 2006 | -0.13(-0.24- -0.01) | 0.03 |
| Change in level in Q1 2007 | -0.14(-0.26- -0.02) | 0.03 |
| Change in level in Q2 2007 | -0.15(-0.28- -0.02) | 0.03 |
| Change in level in Q4 2007 | -0.17(-0.33- -0.02) | 0.03 |
| Change in level in Q2 2008 | -0.20(-0.38- -0.01) | 0.04 |
| **Depression symptoms** |  |  |
| Secular trend | 0.03(-0.01-0.07) | 0.10 |
| Change in level in Q3 2006 | -0.09(-0.37-0.20) | 0.53 |
| Change in level in Q4 2006 | -0.10(-0.39-0.19) | 0.47 |
| Change in level in Q1 2007 | -0.11(-0.42-0.19) | -0.43 |
| Change in level in Q2 2007 | -0.13(-0.46-0.20) | 0.42 |
| Change in level in Q4 2007 | -0.16(-0.56-0.24) | 0.41 |
| Change in level in Q2 2008 | -0.18(-0.67-0.30) | 0.43 |

Supplementary Table S8. Time trend analysis of recording of incident depression diagnosis and symptoms in 15 – 18 year olds in relation to the 2008 recession

| Recording of incident depression | Estimate of Effects (95% CI) | p- value |
| --- | --- | --- |
| **Depression diagnosis** |  |  |
| Secular trend | -0.01(-0.02-0.01) | 0.44 |
| Change in level in Q3 2008 | 0.06(-0.07-0.19) | 0.33 |
| Change in level in Q4 2008 | 0.06(-0.07-0.18) | 0.38 |
| Change in level in Q1 2009 | 0.05(-0.09-0.18) | 0.45 |
| Change in level in Q2 2009 | 0.04(-0.10-0.19) | 0.53 |
| Change in level in Q4 2009 | 0.03(-0.14-0.21) | 0.69 |
| Change in level in Q2 2010 | 0.02(-0.19-0.24) | 0.82 |
| **Depression symptoms** |  |  |
| Secular trend | 0.02(-0.02-0.06) | 0.40 |
| Change in level in Q3 2008 | -0.01(-0.31-0.30) | 0.96 |
| Change in level in Q4 2008 | 0.01(-0.30-0.31) | 0.97 |
| Change in level in Q1 2009 | 0.02(-0.31-0.34) | 0.91 |
| Change in level in Q2 2009 | 0.03(-0.32-0.38) | 0.86 |
| Change in level in Q4 2009 | 0.05(-0.37-0.48) | 0.79 |
| Change in level in Q2 2010 | 0.08(-0.44-0.60) | 0.75 |
